# Long-term healthcare costs and functional outcomes associated with lack of remission in schizophrenia: a post-hoc analysis of a prospective observational study

**DOI:** 10.1186/1471-244X-12-222

**Published:** 2012-12-05

**Authors:** Virginia S Haynes, Baojin Zhu, Virginia L Stauffer, Bruce J Kinon, Michael D Stensland, Lei Xu, Haya Ascher-Svanum

**Affiliations:** 1 Eli Lilly and Company, Lilly Corporate Center, Indianapolis, IN 46285, USA; 2 Agile Outcomes Research, Inc, Rochester, MN, 55902, USA

**Keywords:** Schizophrenia, Health care costs, Prospective studies, Observational studies, Symptom remission, Treatment outcome

## Abstract

**Background:**

Little is known about the long-term outcomes for patients with schizophrenia who fail to achieve symptomatic remission. This post-hoc analysis of a 3-year study compared the costs of mental health services and functional outcomes between individuals with schizophrenia who met or did not meet cross-sectional symptom remission at study enrollment.

**Methods:**

This post-hoc analysis used data from a large, 3-year prospective, non-interventional observational study of individuals treated for schizophrenia in the United States conducted between July 1997 and September 2003. At study enrollment, individuals were classified as non-remitted or remitted using the Schizophrenia Working Group Definition of symptom remission (8 core symptoms rated as mild or less). Mental health service use was measured using medical records. Costs were based on the sites’ medical information systems. Functional outcomes were measured with multiple patient-reported measures and the clinician-rated Quality of Life Scale (QLS). Symptoms were measured using the Positive and Negative Syndrome Scale (PANSS). Outcomes for non-remitted and remitted patients were compared over time using mixed effects models for repeated measures or generalized estimating equations after adjusting for multiple baseline characteristics.

**Results:**

At enrollment, most of the 2,284 study participants (76.1%) did not meet remission criteria. Non-remitted patients had significantly higher PANSS total scores at baseline, a lower likelihood of being Caucasian, a higher likelihood of hospitalization in the previous year, and a greater likelihood of a substance use diagnosis (all p < 0.05). Total mental health costs were significantly higher for non-remitted patients over the 3-year study (p = 0.008). Non-remitted patients were significantly more likely to be victims of crime, exhibit violent behavior, require emergency services, and lack paid employment during the 3-year study (all p < 0.05). Non-remitted patients also had significantly lower scores on the QLS, SF-12 Mental Component Summary Score, and Global Assessment of Functioning during the 3-year study.

**Conclusions:**

In this post-hoc analysis of a 3-year prospective observational study, the failure to achieve symptomatic remission at enrollment was associated with higher subsequent healthcare costs and worse functional outcomes. Further examination of outcomes for schizophrenia patients who fail to achieve remission at initial assessment by their subsequent clinical status is warranted.

## Background

In 2002, the total cost of schizophrenia in the United States was estimated at $62.7 billion, with direct healthcare costs accounting for $22.7 billion, and unemployment accounting for $21.6 billion [1]. Relapse is an important predictor of the direct healthcare costs. Annual average per-patient direct healthcare costs for patients who did or did not experience symptom relapse were $33,187 and $11,771 respectively [2]. Most patients with schizophrenia incur substantial medical costs, are not able to work, and often cannot live independently [1].

Examining the histories of the usual patients with schizophrenia who present for inpatient or outpatient treatment may lead to a universally pessimistic view of the disorder due to selection bias. That is, patients who have very favorable outcomes following initial treatment may be less likely to seek treatment in the future relative to patients who have poor outcomes. Most individuals with schizophrenia function poorly despite treatment; however, long-term studies have documented a favorable course for a subset of patients [3]. A recently published 20-year prospective study reported that most patients with schizophrenia (57%) had persistent or recurring symptoms, but a smaller subset (29%) exhibited no delusions at any of the follow-up assessments [4]. In this smaller subgroup of individuals, those who maintained good functioning even after discontinuing antipsychotic medications were found to have better premorbid functioning, less vulnerability, greater resilience, better self-image, and more favorable prognostic factors than most patients with schizophrenia [5]. Similarly, a review of longitudinal outcomes for first-episode schizophrenia patients, found a subset of patients (42%) had a “good” outcome three years later [6]. Notably, being treated with the combination of antipsychotics and psychosocial treatment was predictive of better outcomes for the first-episode patients [6]. Thus, for a smaller subset of patients with schizophrenia, the long-term course of the disease may be less debilitating.

With the improved understanding of long-term outcomes in schizophrenia and the increasing availability of effective treatment options, the focus on remission in schizophrenia has been growing. An important step occurred in 2005, when the Remission in Schizophrenia Working Group created a consensus definition of symptom remission in schizophrenia [7,8], providing a definition amenable for researching remission in schizophrenia. A growing body of research has linked this definition of remission to several different improved outcomes. In addition to reduced symptoms of schizophrenia [9-18], remitted patients were found to have higher levels of functioning [9,10,19-23], better Health-Related Quality of Life (HRQOL) [9,11,13,22], and reduced healthcare resource use [14]. Because the reduced healthcare resource use was found in a single study in Sweden, more research is needed to identify the implications of failing to achieve remission on healthcare costs.

Despite multiple studies reporting significantly worse clinical and functional status for non-remitted patients, little longitudinal research has investigated the long-term effects of non-remission on healthcare costs and functional outcomes for diverse patients with schizophrenia living in the United States. This post-hoc analysis of a 3-year prospective, observational study compared the costs of mental health services and the functional outcomes between subjects with schizophrenia who met and did not meet cross-sectional symptom remission at study enrollment.

## Methods

This study used data from the U.S. Schizophrenia Case and Assessment Program (US-SCAP), a large (N = 2,327), 3-year prospective, observational study of schizophrenia treatment in usual-care settings that was conducted between July 1997 and September 2003. Data were collected from 41 individual sites in 6 regions (California, Colorado, Connecticut, Florida, Maryland, and North Carolina) throughout the Northeast, Southwest, Mid-Atlantic, and West geographical areas. The sites were intended to be representative of usual care for schizophrenia and included community mental health centers, university health care systems, community and state hospitals, and the Department of Veterans Affairs Health Services. The study was sponsored by Eli Lilly and Company and further details are available elsewhere [2,24,25]. In compliance with the Declaration of Helsinki, the study was approved by Institutional Review Board at each regional site and informed consent was obtained from all participants. The Institutional Review Boards were from the Yale University School of Medicine, Colorado Multiple Institutional Review Board, Children's Hospital in San Diego, University of Maryland at Baltimore, University of South Florida, and Duke University Medical Center.

### Inclusion and exclusion criteria

US-SCAP was designed to capture treatment outcomes for schizophrenia in usual clinical care. The broad inclusion criteria required patients to be at least 18 years of age and diagnosed with schizophrenia, schizoaffective, or schizophreniform disorders based on the criteria of the Diagnostic and Statistical Manual of Mental Disorders, Version 4 (DSM-IV) [26]. Enrollment was not contingent upon the use of any particular medication, concurrent psychiatric or medical conditions, the use of concomitant medications, or the presence of substance abuse. Participants of the US-SCAP study could stay on medications received prior to enrollment. All treatment decisions, including any medication changes were made by the treating physicians and patients. Participants were excluded only if they were unable to provide informed consent, unlikely to be accessible for follow-up visits, or if they had participated in a clinical drug trial within 30 days prior to enrollment.

### Measures

Outcome measures in this study were grouped into four basic categories: symptoms of schizophrenia; healthcare resource utilization and costs; HRQOL and functioning; and violence, victimization, and arrests. The results and discussion were organized accordingly.

#### Symptoms of schizophrenia

Symptoms of schizophrenia were captured using the Positive and Negative Syndrome Scale (PANSS) [27]. The PANSS is a 30-item, clinician-rated measure of common symptoms of schizophrenia. Each item was rated on a 1–7 scale with higher numbers representing more severe symptoms. The range for the PANSS total score was from 30 to 210. The PANSS was administered at baseline and then annually. In addition, a PANSS symptoms of remission (PANSS-SR) subscale was created by summing the eight core symptom items used to define remission (see below). The range for the PANSS-SR subscale was from 8 to 56.

#### Healthcare resource utilization and costs

A Medical Record Abstraction Form (MRAF) was developed specifically for this study to collect information from the patients’ healthcare records including diagnoses, medication use, individual therapy, group therapy, rehabilitation and mental health-related outpatient services, and inpatient services. Comorbid substance use, mental retardation, and personality diagnoses were identified based on the information collected from patients’ medical records and recorded in the MRAF. The medical records were abstracted at baseline and then at 6-month intervals by trained examiners. Implementation of the MRAF was limited at the beginning of the study resulting in missing data for some of the early participants. Patients were also queried about treatments they received outside of their usual healthcare sites and study personnel obtained medical records from these sites as needed.

Costs were calculated based on the MRAF information reported at the time of service. Due to variations across sites, the costs of mental health services other than psychiatric hospitalizations were based on their Medicare relative value units developed from data management information systems at each site. Hospitalization costs were calculated as $556 per day, which was the average hospitalization per-diem charge across study sites. Hospital or inpatient costs included any overnight stay at a hospital including both community-based hospital beds and long-term psychiatric beds. Medications were priced based on Average Wholesale Price discounted by 15% to reflect the customary discount level in the United States. All costs not attributed to medications, emergency rooms, or hospitalizations were considered outpatient costs. All costs were based on the year 2000, the mid-year of the US-SCAP study. The cost outcome variables examined in this study included total costs, hospitalization costs, emergency room costs, total medication costs, and antipsychotic medication costs.

The Schizophrenia Care and Assessment Program Health Questionnaire (SCAP-HQ) [24] included questions relevant to healthcare resource use. Patients were asked about the number of overnight stays in the hospital for mental or emotional problems as well as any emergency visits with psychiatrists and therapists in the past 4 weeks. The SCAP-HQ also included a measure of non-adherence for psychiatric medications during the past 4 weeks. Scores ranged from 1 to 5, with higher scores indicating worse medication adherence.

#### HRQOL and functioning

The Quality of Life Scale (QLS) [28] is a 21-item, clinician-rated scale assessing symptoms and functional status during the previous 4 weeks. QLS items are rated on a 0–6 scale with higher numbers representing more normal levels of functioning. The QLS total scores could range from 0 to 126. The QLS measure includes four subscales: Intrapsychic Foundations (7 items; subscale range 0–42), Interpersonal Relations (8 items; subscale range 0–48), Instrumental Role (4 items; subscale range 0–24), and Common Objects and Activities (2 items; subscale range 0–12).

Medical Outcomes Survey 12-item Short Form Health Survey (SF-12) [29] is a generic measure of HRQOL that gives two summary scores: Mental Component Summary (MCS) and Physical Component Summary (PCS). The scores have been normalized to yield a mean of 50 and a standard deviation of 10 based on the U.S. population with higher scores indicating better functioning.

The Global Assessment Functioning Scale (GAF) [26] is an anchored clinician rating of patient functioning that is part of the DSM-IV multiaxial diagnostic assessment. Scores range from 1 to 100, with 100 representing superior functioning.

The SCAP-HQ [24] included several simple measures of functioning. At each assessment, patients reported their current living status, which was scored as living independently (“yes” or “no”). Patients also reported if they worked at a job for pay during the past 4 weeks (“yes” or “no”). Finally, patients’ reported their satisfaction with meeting basic needs and their general life satisfaction during the past four weeks. These two satisfaction measures were each scored from 1 to 7 with higher scores indicating greater satisfaction.

#### Victimization, violence, and arrests

The SCAP-HQ included several straightforward measures of possible involvement with the criminal justice system. Victimization was based on patients’ self-reports of whether or not they were victims of a crime during the past four weeks. Violence was based on patients’ self-reports of striking or injuring anyone during the past four weeks. Finally, arrests were based on patients’ self-reports of being arrested during the past 6-months. All of these measures were scored as “yes” or “no.”

### Definition of remission

Remission was based on the Remission in Schizophrenia Working Group definition [7]. Participants were classified as remitted if their symptoms were rated as mild, minimal, or absent on eight core items of the PANSS: delusions (P1), unusual thought content (G9), hallucinatory behavior (P3), conceptual disorganization (P2), mannerisms/posturing (G5), bunted affect (N1), social withdrawal (N4), and lack of spontaneity (N6). The current study defined remission based only on symptoms at baseline and did not use the 6-month duration requirement.

### Statistical methods

Differences in baseline characteristics between non-remitted and remitted patients were tested with chi-square tests for categorical variables and t-tests for continuous variables. For continuous outcome measures, the differences between non-remitted and remitted patients were assessed using mixed effects models for repeated measures (MMRM) with visit, baseline remission, and the visit by baseline remission interaction as the fixed effects and multiple baseline variables as the covariates. Baseline covariates were age, race, gender, education level, marital status, prior hospitalization, illness duration, schizoaffective diagnosis, substance use diagnosis, personality disorder diagnosis, mental retardation diagnosis, and insurance type. For categorical outcome measures, differences between non-remitted and remitted patients were assessed using a general estimating equation with an exchangeable working correlation matrix, terms for visit, baseline remission, visit by baseline remission interaction, and the same set of baseline covariates as used for the MMRM.

The table and graphs for this study display the observed means and standard deviations or percentages. With the exception of the cost measure, the number of observations at the baseline, 1-, 2-, and 3-year follow-up visits were 1738, 1300, 1117, and 898, respectively for the non-remitted patients and 546, 461, 419, and 330, respectively for the remitted patients. Sensitivity analyses were conducted on total costs with and without using multiple imputation to account for the missing data. The significance level was set at α = 0.05. All analyses were completed using SAS version 9.1 (SAS Institute, Cary, NC).

## Results

### Sample description

About half of the patients (53.8% or 1228 of 2284) completed the 3-year study. The majority of the 2,284 patients in the sample did not meet the criteria for remission at enrollment (n = 1,738; 76.1%), while 23.9% patients did meet remission criteria. The comparisons between non-remitted and remitted patients at baseline are presented in Table 1. Patients who did not meet the criteria for remission were more likely to be male, black, less educated, single, and have a more severe clinical profile at baseline. Their overall HRQOL was lower than remitted patients.

**Table 1:** Baseline Characteristics of Non-Remitted and Remitted Patients

**Characteristic**	**Non-Remitted N = 1738**	**Remitted N = 546**	**P-value**
Age, mean (SD)	41.7 (10.9)	42.1 (12.1)	0.477
Age at Onset, mean (SD)	19.9 (8.8)	21.2 (9.0)	0.003
Duration of Illness, mean (SD)	21.6 (11.9)	20.6 (11.2)	0.093
Gender (Male), %	63.0	57.3	0.019
**Race, %**			0.002
White	47.9	54.6	
Black	38.0	29.9	
Others	14.0	15.6	
Education (< High School), %	69.4	64.5	0.032
Marital Status (Never Married), %	63.0	57.0	0.012
**Insurance Type, %**			0.021
Medicaid/Medicare	82.5	79.9	
VA	5.8	5.0	
Private	3.6	5.7	
No Coverage	6.8	9.0	
Others	1.3	0.4	
Schizoaffective Diagnosis, %	32.1	39.0	0.003
Substance Use Diagnosis, %	28.7	23.6	0.022
Personality Disorder Diagnosis, %	15.2	12.3	0.091
Mental Retardation Diagnosis, %	10.1	6.0	0.004
Hospitalized in Prior Year, %	41.4	32.8	<0.001
PANSS Total Score, mean (SD)	76.1 (16.7)	52.1 (10.3)	<0.001

### Longitudinal comparisons

#### Symptoms of schizophrenia

**Figure 1:**
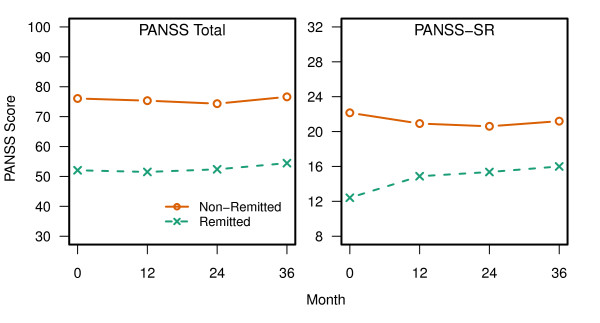
**PANSS Total Score and PANSS-SR.** PANSS total scores were significantly higher for non-remitted than remitted patients across the 3-year period (p < 0.001). In addition, there was a significant effect for visit (p = 0.002). No significance tests were conducted between non-remitted and remitted patients on the PANSS-SR because these scores were used to define remission status at baseline.

PANSS Total scores were significantly higher for the non-remitted patients across the 3-year study (see Figure 1). The significant effect for visit indicated that the PANSS Total scores changed over time. PANSS-SR scores across time are also presented in Figure 1. No significance tests were conducted because these items were used to define remission status.

#### Healthcare resource use and costs

**Figure 2:**
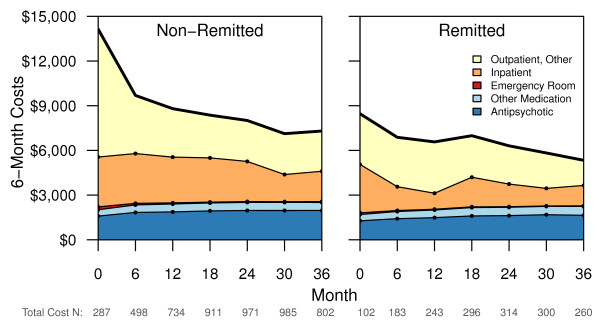
**Healthcare Costs.** The stacked line figure displays the average healthcare costs reported at each visit. The sum of each cost component adds up to the total costs represented by the dark line. The total healthcare, emergency room, total medication, and antipsychotic costs were significantly higher for the non-remitted than remitted patients across the 3-year study (all p < 0.05). There was a significant visit effect for total costs, inpatient costs, emergency room costs, total medication, and antipsychotic costs (all p < 0.05). Finally there was a significant baseline remission status by visit interaction for total and emergency room costs (p < 0.05). Outpatient, Other was a heterogeneous category made up of the remaining costs and was not explicitly modeled.

Total healthcare costs were contrasted between the non-remitted and remitted patients for each 6-month period during the 3-year study. In addition, the following cost categories were compared between the non-remitted and remitted patients: antipsychotic costs, total medication costs, emergency room costs, and inpatient costs. Figure 2 displays these costs at each of the 6-month periods during the study.

A sensitivity analysis using multiple imputation of the missing data confirmed the conclusion of differences in total costs between remitted and non-remitted patients. The difference in emergency room costs over the 3-year period was confirmed using the patients’ self-report measure on the SCAP-HQ that did not have the missing values for the early patients in the study.

Medication non-adherence was significantly worse for the non-remitted patients compared to those who obtained remission at baseline (p < 0.001). The non-remitted patients rated their non-adherence as 1.48, 1.43, 1.39, and 1.37 at the baseline, 1-year, 2-year, and 3-year visits, whereas the remitted patients rated their non-adherence significantly lower across the 3-year study as 1.36, 1.33, 1.30, and 1.30 at the corresponding visits (p < 0.001). There was a significant visit effect (p = 0.012), but the visit by remission baseline interaction was not significant (p = 0.937), indicating a similar pattern of decline in adherence for both groups.

#### HRQOL and functioning

**Figure 3:**
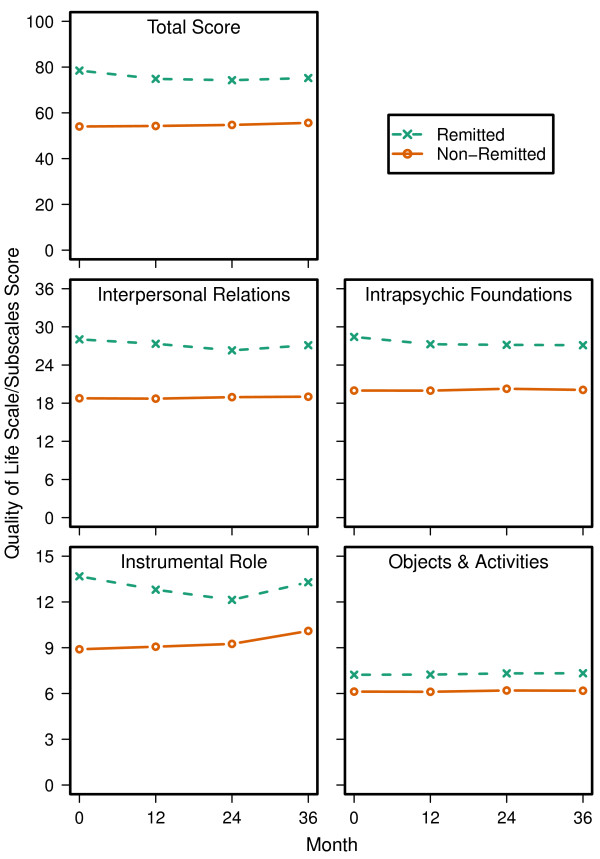
**QLS Total and Subscale Scores.** QLS total scores and each of the QLS subscale scores were significantly lower for non-remitted than remitted patients across the 3-year period (p < 0.001). In addition, there was a significant effect for visit (p < 0.05) and a significant interaction between baseline remission and visit (p < 0.01) on the total score and all of the QLS subscales except for Common Objects and Activities.

**Figure 4:**
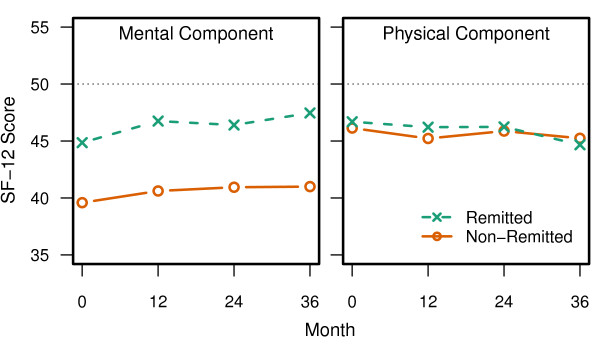
**MOS SF-12.** Non-remitted patients had significantly lower Mental Component Scores across the 3-year study (p < 0.001). Physical Component Scores were not significantly different (p = 0.325). For both of these scales there was a significant effect for visit (p < 0.01). The dotted line at 50 represents the average score for the US population.

**Figure 5:**
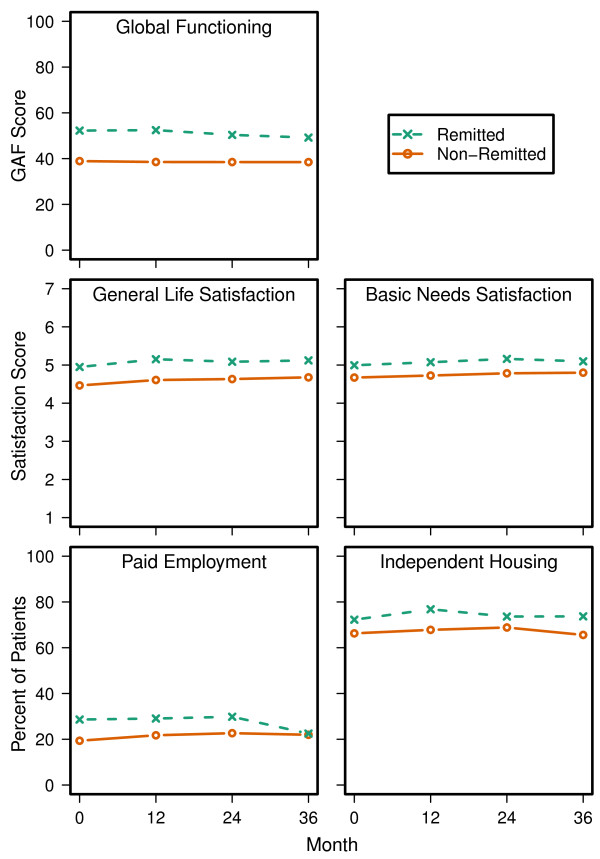
**Functional Measures.** Across the 3-year study period, non-remitted patients scored significantly lower on the GAF, Satisfaction with Fulfilling Basic Needs, and the General Life Satisfaction measures than remitted patients (p < 0.001). Additionally, non-remitted patients were less likely to have Paid Employment across the 3-year study (p < 0.01), but no significant main effect was found for Independent Housing status. A significant visit effect (p < 0.05) was found for the GAF, Satisfaction with Fulfilling Basic Needs, General Life Satisfaction, and for Independent Housing status. There was a significant baseline remission by visit interaction for the GAF and Independent Housing status (p < 0.05).

On multiple measures of HRQOL and functioning, the non-remitted patients had greater impairment across all 3 years of the study. The details of these results are presented with multiple figures: Quality of Life Scale in Figure 3, SF-12 in Figure 4, GAF, General Life Satisfaction and Satisfaction with Fulfilling Basic Needs, and Paid Employment and Living Independently in Figure 5. On all of the measures of HRQOL and functioning, with the exception of Living Independently and SF-12 PCS, non-remitted patients had significantly worse functioning and quality of life across the 3-year study.

#### Victimization, violence, and arrests

**Figure 6:**
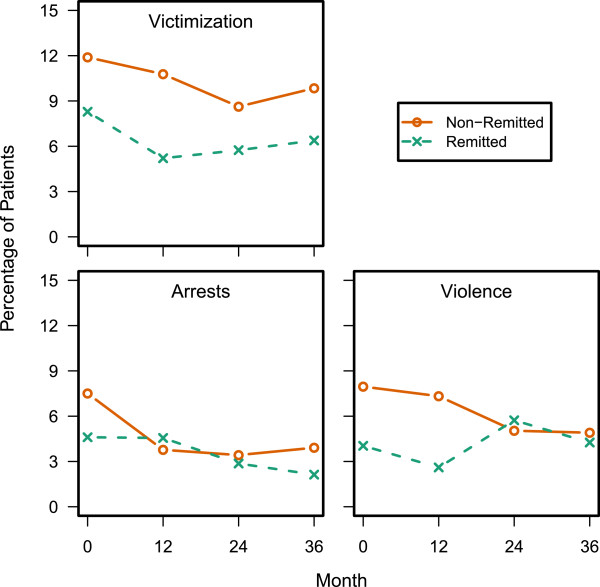
**Victimization, Violence, and Arrests.** Non-remitted patients reported a greater likelihood of being victimized or committing a violent act than remitted patients during the 3-year study (p < 0.05). For victimization and arrests there was a significant effect for visit (p < 0.05). Additionally, there was a baseline remission by visit interaction for violence (p = 0.019).

Figure 6 displays the observed differences between non-remitted and remitted patients on Violence, Victimization, and Arrests. Across the 3-year study, non-remitted patients were significantly more likely to report being the victims of crimes or perpetrating violence.

## Discussion

In this large, geographically and clinically diverse sample of US patients with schizophrenia, 23.9% met the criteria for symptom remission at baseline. This post-hoc analysis of a 3-year prospective, observational study demonstrated that failure to achieve remission at study enrollment was associated with increased symptoms of schizophrenia, increased healthcare costs, worse HRQOL and functional outcomes, and a greater likelihood of interacting with the criminal justice system. Even though remission status was defined at baseline, the differences for most measures appeared stable across all 3 years of the study.

Consistent with past research, this study found non-remitted patients had more severe symptoms of schizophrenia [9-18,30]. Given that remission is defined based on the symptom rating, this finding was expected at baseline. More informative was the finding that the difference in symptoms largely remained across the 3-year study. This finding replicates an earlier study [9] which found non-remitted patients to continue to be more symptomatic than remitted patients 3 years later. The subset of patients who met remission criteria appeared to maintain lower levels of symptoms over time.

Non-remitters had significantly higher costs in every category but inpatient costs. Although the costs decreased over time, differences remained between the baseline non-remitters and remitters. After baseline, the total cost difference ranged between $1200 and $2800 greater for the non-remitted patients during every 6-month period. This finding appears to be a unique contribution to the literature. Prior cross-sectional research in Sweden found patients who obtain remission use fewer healthcare services, although this was not linked directly to costs [14]. Effective treatments that move patients into remission could potentially reduce the burden of schizophrenia on the healthcare system, but more research is needed.

The non-remitted patients reported worse medication adherence. Some of the increased costs could be due to reduced medication adherence resulting in more relapses [31]. Relapses have substantial effects on healthcare costs [2] and medication adherence has been previously shown to be associated with remission [32].

For multiple clinician and patient-rated measures of HRQOL and functioning, the non-remitted patients appeared significantly more impaired at baseline and across the 3-year study. This was found for all studied measures except the physical component score of the SF-12 and the percentage of patients living independently. Significance tests showed that some of the functional measures were changing over time for the non-remitted or remitted patients, but the time effects were small relative to the effect of symptom remission. Worse functioning and quality of life in non-remitted patients has been reported in past research [9-11,13,19-23]. On the SF-12 summary scores, the remitted patients average score was below the population average score of 50. This highlights that meeting the criteria for symptom remission does not imply clinical recovery in schizophrenia.

Recovery is schizophrenia has been defined objectively as clinical recovery or subjectively as personal recovery [33,34]. Clinical recovery, which has been the focus in the scientific literature, defines recovery as the absence of symptoms and returning to levels of premorbid functioning including working, living independently, and carrying out activities of daily living [33]. Personal recovery focuses on the more subjective process of adaption to the illness and encompasses self-awareness, a sense of empowerment, and functioning at one’s best despite ongoing symptoms [34,35]. Important concepts in personal recovery include overcoming poverty, stigma, demoralization, hopelessness, and social isolation [35]. Recent research has found that the development of a personal narrative mediates the relationship between deficits in social cognition or social withdrawal and negative symptoms [36] and that vocational rehabilitation is linked to reductions in self-stigma [37]. Future research is needed to examine the association between symptom remission and measures of personal recovery. Whether considered from the clinical or personal perspective, recovery in schizophrenia is the ultimate goal and goes beyond symptom remission [33,34].

The current study contained a unique set of variables asking patients about past violence, victimization and arrests. Although the overall incidence for each was low, and appeared to decrease slightly over the 3-year study, non-remitted patients were significantly more likely to report violent behaviors as well as being victims of crimes than the remitted patients across the 3-year study. The difference in violent behaviors was more prominent in the first year of the study. Further research into the potential legal repercussions of failing to obtain remission is needed. Individuals with schizophrenia appear to be at an increased risk for repeat incarcerations [38].

The findings of this study demonstrated that over a 3-year period, non-remitted patients have a substantially increased burden on the United States healthcare system compared to patients who have obtained baseline remission. Although reduced healthcare use has been shown previously in Sweden [14], the current study extends this finding to costs over three years among a large representative sample of individuals with schizophrenia in the US. Potential healthcare savings of moving patients into remission could be as high as $1200 to $2800 per patient every six months. Perhaps, treating schizophrenia more aggressively with more efficacious agents [39] or combination therapy [40] could result in more patients reaching remission and reduce the economic burden on the healthcare system, but more research is clearly needed.

Alternatively, remission status in schizophrenia may represent a patient “trait” characteristic rather than a current “state.” Past research has identified certain patient characteristics that are predictive of obtaining symptom remission in schizophrenia: higher educational status [12], lower symptoms severity [12,32], being married [12], shorter duration of untreated psychosis [12], no substance use diagnosis [32], and higher levels of functioning (employed, living independently, and higher subjective well being under neuroleptics scores) [32]. Several of these same variables were significantly different between the remitted and non-remitted patients at baseline in the current study (see Table 1). Constructs from personal recovery in schizophrenia, such as a sense of personal agency, may have also differed between the remitted and non-remitted patients [41], but these were not measured in our study. Achieving symptom remission may reflect characteristics of certain patients with schizophrenia who tend to have favorable outcomes rather than the effects of treatment. On the other hand, initial treatment with atypical instead of typical antipsychotics has been predictive of achieving symptom remission [32] and treating first-episode patients with both antipsychotic and psychosocial treatment has been predictive of better long-term outcomes [6]. More research is needed to differentiate the patient selection effects from the treatment effects on symptom remission.

### Limitations

In this study, remission was defined at baseline only, but the published criteria also require the reduced symptoms to be maintained for a period of at least 6 months [7]. Had the longitudinal requirement been added, some patients classified as remitted may have been classified as non-remitted. However, the US-SCAP study only collected the PANSS annually and the consistent differences between the two cohorts on multiple measures over time suggest that most of those classified as remitted likely stayed in remission. In addition, the results do not provide information about gains from non-remitted patients who subsequently reached treatment remission. Instead, this study can only provide information about the differences between those who were classified as remitted or non-remitted at baseline. In this study, a substantial rate of missing data for the total costs might have led to unreliable estimates of cost differences between remitted and non-remitted patients. Nevertheless, sensitivity analysis using multiple imputation to impute the missing data confirmed the overall findings. Finally, the label of remission does not mean complete functional recovery. In this study, patients meeting the criteria for remission continued to display functional impairments and did not achieve functional levels of the general population.

## Conclusions

In this post-hoc analysis of a 3-year prospective observational study, the failure to achieve symptomatic remission at enrollment was associated with higher subsequent healthcare costs and worse functional outcomes. Further examination of outcomes for patients who move from non-remission into remission is warranted.

## Competing interests

The authors, Virginia Haynes, Baojin Zhu, Virginia Stauffer, Bruce Kinon, Lei Xu, and Haya Ascher-Svanum, are full-time employees and minor stockholders of Eli Lilly and Company or its subsidiaries. Michael D. Stensland is a full-time employee of Agile Outcomes Research, Inc, a contract research organization that was hired by the sponsor.

## Authors’ contributions

VSH, BZ, VLS, BJK, MDS, LX, and HA-S contributed to the conception and design of the study. BZ and LX performed the statistical analyses. All authors helped draft the manuscript and approved the final version.

## Pre-publication history

The pre-publication history for this paper can be accessed here:

http://www.biomedcentral.com/1471-244X/12/222/prepub
